# D-dimer, a predictor of bad outcome in gastric cancer patients undergoing radical resection

**DOI:** 10.1038/s41598-022-16582-9

**Published:** 2022-09-30

**Authors:** Xin Zhang, Xuan Wang, Wenxing Li, Tuanhe Sun, Chengxue Dang, Dongmei Diao

**Affiliations:** Department of Surgical Oncology, First Affiliated Hospital Medical College Xi’an Jiaotong University, 277 West Yanta Road, Xi’an, Shaanxi 710061 People’s Republic of China

**Keywords:** Cancer, Biomarkers, Risk factors

## Abstract

As a marker of hypercoagulability, plasma D-dimer is associated with progression of many cancers but remains controversial in gastric cancer (GC). We aim to investigate the predictive value of D-dimer for postoperative outcomes after radical gastrectomy of GC patients. We enrolled 903 consecutive patients with GC who underwent radical gastrectomy and the clinicopathological characteristics were compared. Risk factors for overall survival (OS) and disease-free survival (DFS) were determined using multivariate cox regression analysis. We also compared the survival difference based on Kaplan–Meier method after a one-to-one propensity score matching (PSM). Patients with elevated D-dimer had older age (p < 0.001), advanced TNM stage (p < 0.001), larger tumor size (p = 0.005), lower 5-year OS rate (32.8% vs 62.6%, p < 0.001) and DFS (29% vs 59.6%, p < 0.001). In multivariate analysis, elevated D-dimer was independently associated with shorter OS [hazard ratio (HR): 1.633, 95% confidence interval (CI) 1.178–2.264, p = 0.003] and DFS (HR: 1.58, 95% CI 1.151–2.169, P = 0.005). After PSM, the 5-year OS rate of patients with elevated D-dimer was still significantly lower than matched group (32.8% vs 40.6%, p = 0.005), so was DFS (29% vs 36.6%, p = 0.008). Preoperative elevated D-dimer is an independent risk factor for GC patients undergoing curative gastrectomy.

## Introduction

As a global health threat, approximately one million people worldwide are newly diagnosed with gastric cancer each year. Gastric cancer remains the fifth most frequently diagnosed disease and the third leading cause of cancer-related death, despite a decline in morbidity and mortality worldwide over the past 50 years^1^. An estimated 4.3 million new cancer cases and 2.9 million new cancer deaths occurred in China in 2018^2^.Gastric cancer is the second leading cause of cancer death in men and women in China. An estimated 679,100 new cases of gastric cancer are diagnosed in China each year^3^. As more than 80% of patients diagnosed at an advanced stage, 5-year survival rate of gastric cancer still remains very low^4^.

Hypercoagulability is frequently observed in several tumors, which not only closely related to thrombosis, but also associated with tumor progression. D-dimer, as a degradation product of fibrin, is produced when cross-linked fibrin is degraded by plasmin induced fibrinolytic activity. D-dimer have been used as a screening and diagnostic tool in numerous coagulopathies and thrombotic disease. To date, a growing number of reports have demonstrated that hemostatic abnormalities are closely related to cancer^5^, such as lung^6,7^, breast^8,9^, esophageal^10^, colorectal^11^, and gastric cancer^12^. Many studies confirmed that high D-dimer levels may predict poor outcomes for GC patients^13–16^. Consistently, our previous research showed that D-dimer is not only an indicator of venous thrombosis but also a marker for predicting cancer progression in GC patients^17^. In contrast, Liang et al. reported that elevated preoperative D-dimer was not an independent prognostic factor for GC based on propensity score matching analysis^18^. Based on this controversy, the aim of our study was to explore the correlation between preoperative D-dimer and long-term survival of GC patients after curative surgery. In order to improve credibility, we collected data from three medical centers. Taking into consideration that baseline characteristics may influence the accuracy of prognostic analysis of D-dimer, we conduct analysis on both whole series and matching series based on COX proportional hazard model and propensity score method, respectively.

## Results

### Demographic and clinicopathological features of two patient groups before PSM

**Figure 1:**
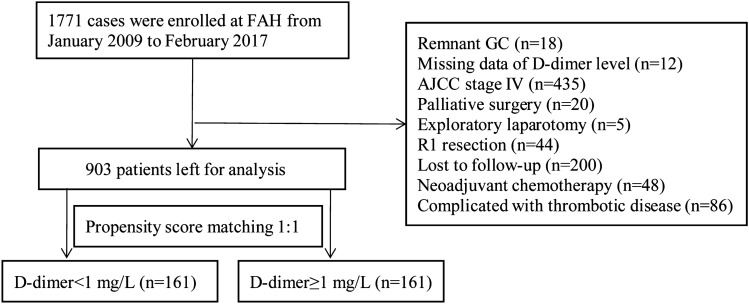
Flowchart with a summary of patient enrollment and propensity score matching. FAH, the First Affiliated Hospital of Xi'an Jiaotong University.

We retrospectively enrolled 1771 consecutive cases and 903 remained for analysis after excluding 868 patients according to exclusion criteria (18 cases of gastric stump cancer, 12 cases with D-dimer data missing, 435 cases at stage IV, 20 cases treated with palliative surgery, 5 cases treated with exploratory laparotomy, 44 cases with R1 resection, 48 cases treated with neoadjuvant chemotherapy before surgery, 86 cases concurrent with thrombotic disease, 113 patients refused to surgical treatment and 87 cases lost to follow-up) (Fig. 1). Among all patients, 687 were (76.1%) men and 290 (32.1%) patients were over 65 years old. To assess the relationship between hypercoagulability and disease progression, the whole series were divided into NDG group (D-dimer < 1 mg/l, n = 742) and EDG group (D-dimer ≥ 1 mg/l, n = 161) according to the upper limit of normal value of clinical reference of D-dimer. Patients in EDG had a higher proportion of female (30.4% vs 22.5%, p = 0.041), higher INR(median: 1.03 vs 1.02, p = 0.012), FIB (median: 3.44 g/l vs 3.12 g/l, p < 0.001), FDP (median: 3.2 mg/l vs 0.9 mg/l, p < 0.001), more advanced TNM stage (p < 0.001), higher LNR (median: 0.21 vs 0.05, p < 0.001), larger tumor size (median: 4.5 cm vs 4 cm, p = 0.005), shorter OS (median: 19 months vs 39 months, p < 0.001) and shorter DFS (median: 18.5 months vs 39 months, p < 0.001) (Table 1). No significant difference was found in PT, PTA, PTR, TT, APTT, tumor location and histology. Correlations of D-dimer and other clinicopathologic features was listed. Spearman correlation analysis demonstrated a greatly relationship between plasma D-dimer and age (P < 0.001), tumor size (P = 0.002), T stage (P = 0.001), N stage (< 0.001) and TNM stage (P < 0.001) (see Supplementary Table S1 online).

**Table 1:** Demographic and baseline characteristics of the two patient groups before PSM (N = 903).

Characteristics	Total	NDG	EDG	P value
	(N = 903)	(N = 742)	(N = 161)	
**Gender, no. (%)**				0.041
Female	216(23.9)	167(22.5)	49(30.4)	
Male	687(76.1)	575(77.5)	112(69.6)	
Age ≥ 65 years, no. (%)	290(32.1)	211(28.4)	79(49.1)	< 0.001
PT, second	13(12.5–13.5)	13(12.5–13.5)	13.1(12.6–13.7)	0.091
PTA, %	96.46 ± 17.25	96.91 ± 16.95	94 ± 18.67	0.096
PTR	1.02(0.98–1.07)	1.02(0.98–1.07)	1.02(0.99–1.08)	0.156
APTT, second	34.6(32–37.2)	34.55(32.1–37.33)	34.7(31.55–36.95)	0.382
INR	1.02(0.98–1.07)	1.02(0.98–1.07)	1.03(1–1.09)	0.012
TT, second	16.3(15.7–16.9)	16.3(15.8–16.9)	16.2(15.6–16.85)	0.333
FIB, g/l	3.2(2.64–3.8)	3.12(2.62–3.7)	3.44(2.81–4.2)	< 0.001
D-dimer, mg/l	0.5(0.2–0.9)	0.4(0.1–0.6)	1.7(1.2–2.75)	< 0.001
FDP, mg/l	1.1(0.6–1.83)	0.9(0.6–1.4)	3.2(2.3–5.4)	< 0.001
Platelet	202(157–254)	202.5(157–255)	200(155.8–250.5)	0.77
**Tumor location, no. (%)**				0.992
Proximal stomach	294(32.6)	241(32.4)	53(32.9)	
Distal stomach	478(52.9)	393(53)	85(52.8)	
Total stomach	131(14.5)	108(14.6)	23(14.3)	
**T stage, no. (%)**				< 0.001
T1	190(21)	172(23.2)	18(11.2)	
T2	76(8.4)	68(9.2)	8(5)	
T3	153(16.9)	114(15.4)	39(24.2)	
T4	484(53.6)	388(52.3)	96(59.6)	
**N stage, no. (%)**				< 0.001
N0	385(42.6)	334(45)	51(31.7)	
N1	143(15.8)	122(16.4)	21(13)	
N2	166(18.4)	138(18.6)	28(17.4)	
N3	209(23.1)	148(19.9)	61(37.9)	
**TNM stage, no. (%)**				< 0.001
I	223(24.7)	199(26.8)	24(14.9)	
II	149(16.5)	130(17.5)	19(11.8)	
III	531(58.8)	413(55.7)	118(73.3)	
**Chemotherapy**				0.053
Yes	546(60.5)	456(61.5)	112(69.6)	
No	335(37.1)	286(38.5)	49(30.4)	
**Histology, no. (%)**				0.78
Differentiated	289(32)	236(31.8)	53(32.9)	
Undifferentiated	614(68)	506(68.2)	108(67.1)	
OS, month	37(19–59)	39(24–61)	19(10–42.5)	< 0.001
DFS, month	37(18–59)	39(23–61)	18.5(9–43.25)	< 0.001
LNR	0.07(0–0.31)	0.05(0–0.28)	0.21(0–0.56)	< 0.001
Tumor size, cm	4(2.5–5.5)	4(2.5–5.5)	4.5(3–6)	0.005

Abbreviations: PSM, Propensity Score Matching; NDG, normal D-dimer group (< 1 mg/l); EDG, elevated D-dimer group (≥ 1 mg/l); PT, prothrombin time; PTA, prothrombin activity; PTR, prothrombin ratio; APTT, activated partial prothrombin time; INR, international normalized ratio; TT, thrombin time; FIB, fibrinogen; FDP, fibrin degradation products; TNM, Tumor-node-metastasis; OS, overall survival; DFS, disease free survival; LNR, Lymph node ratio.

### Plasma D-dimer is an independent risk factor for GC survival

**Figure 2:**
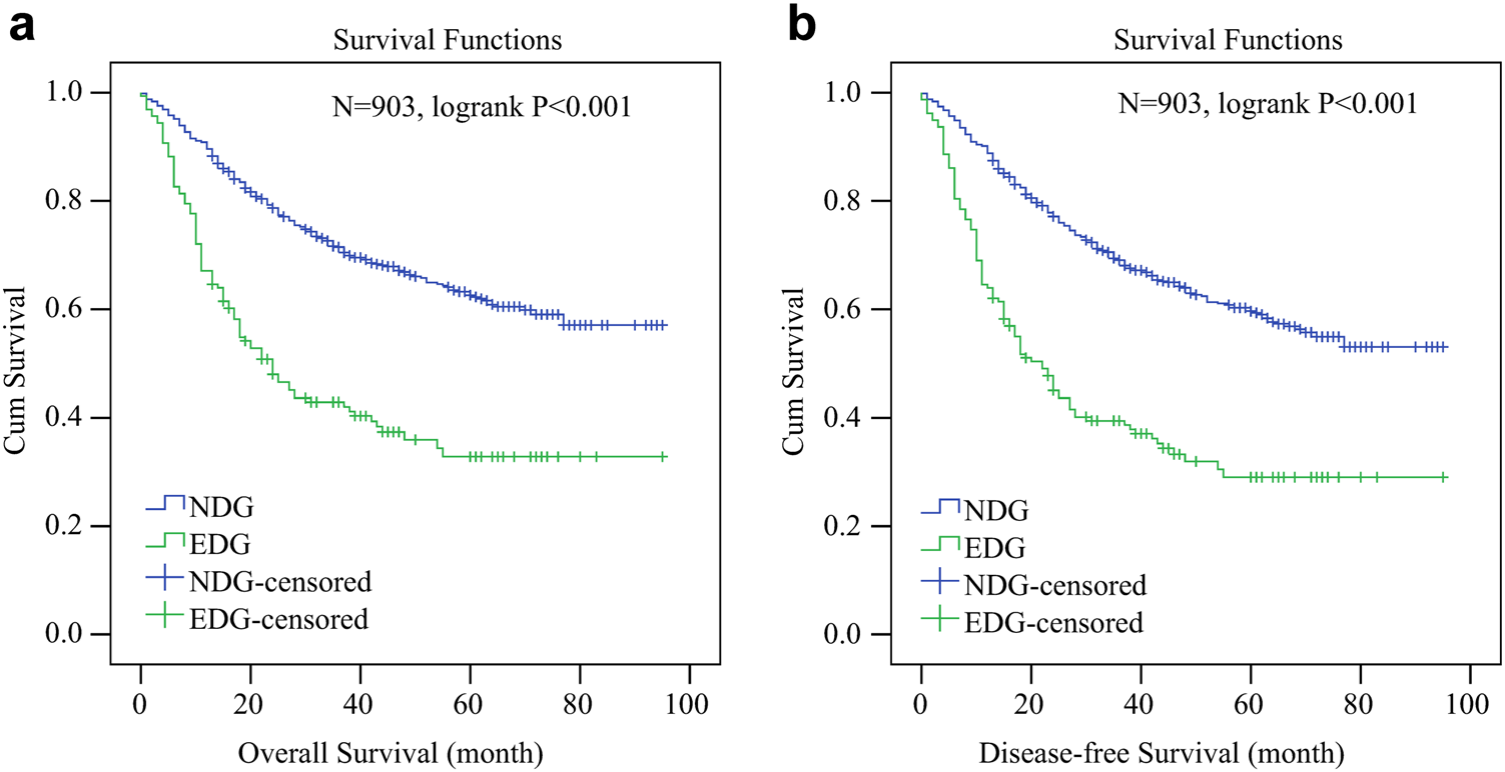
Kaplan–Meier (KM) survival curves for whole GC patient series who underwent radical gastrectomy (N = 903). (**a**) the 5-year overall survival rate was 62.6% for NDG and 32.8% for EDG (P < 0.001). (**b**) the 5-year disease-free survival rate was 59.6% for NDG and 29% for EDG (P < 0.001).

**Figure 3:**
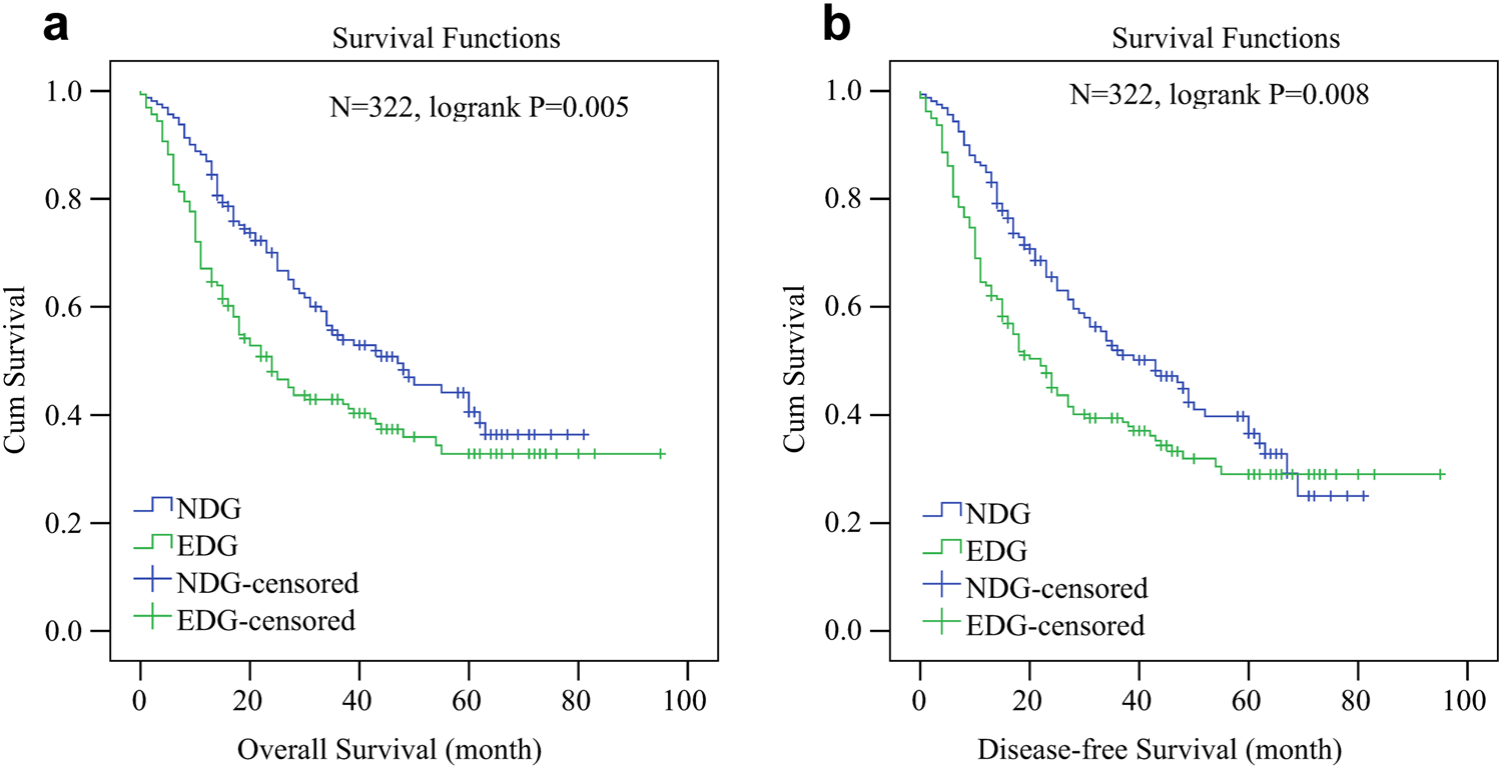
Kaplan–Meier (KM) survival curves for matched GC patient series who underwent radical gastrectomy (N = 322). (**a**) the 5-year overall survival rate was 40.6% for NDG and 32.8% for EDG (P = 0.005). (**b**) the 5-year disease-free survival rate was 36.6% for NDG and 29% for EDG (P = 0.008).

The optimal cutoff value of PT, PTA, PTR, INR, APTT, TT, FIB, D-dimer and FDP determined by ROC analysis was 13.2, 102.6, 1, 0.96, 38.9, 16.5, 3.8, 1 and 2.1, respectively (Supplementary Table S2, Supplementary Fig. 1–3). In univariate COX regression analysis, elder age, distal stomach (proximal stomach for reference), higher coagulation indicators (PTA, INR, TT, FIB, D-dimer and FDP), higher LNR, larger tumor size and advanced TNM stage were significantly associated with shorter OS. In multivariate analysis, age (HR: 1.918, 95% CI 1.436–2.561, P < 0.001), D-dimer (HR: 1.633, 95% CI 1.178–2.264, P = 0.003), LNR (HR: 4.707, 95% CI 2.886–7.677, P < 0.001), tumor size (HR: 1.085, 95% CI 1.032–1.141, P = 0.002), adjuvant chemotherapy (HR: 0.42, CI%: 0.286–0.615, P < 0.001) and TNM stage (TNM II stage: HR: 4.063, 95% CI 1.897–8.705, P = 0.001, TNM III stage: HR: 9.296, 95% CI 4.669–18.51, P < 0.001) were independent prognostic factors for OS (Table 2). The 5-year OS rate in EDG was markedly lower than in NDG (32.8% vs 62.6%, p < 0.001) (Fig. 2A). Independently prognostic factors for DFS were the same as for OS: age (HR:1.914, 95% CI 1.448–2.531, P < 0.001), D-dimer (HR: 1.58, 95% CI 1.151–2.169, P = 0.005), LNR (HR: 4.865, 95% CI 3.037–7.793, P < 0.001), tumor size (HR: 1.084, 95% CI 1.034–1.141, P = 0.001), chemotherapy (HR: 0.434, CI%: 0.3–0.63, P < 0.001) and TNM stage (TNM II stage: HR: 3.615, 95% CI 1.792–7.293, P < 0.001, TNM III stage: HR: 7.803, 95% CI, 4.136–14.722, P < 0.001) (see Supplementary Table S3 online). Significant difference was found between NDG and EDG for 5-year DFS rate (29% vs 59.6%, p < 0.001) (Fig. 3A).

**Table 2:** Univariate and multivariate analyses for overall survival of GC patients before PSM (N = 903).

Parameters	Univariate analysis			Multivariate analysis		
	HR	95% CI	P value	HR	95% CI	P value
Gender	1.181	0.918–1.519	0.196			
Age	1.72	1.389–2.13	< 0.001	1.918	1.436–2.561	< 0.001
**Tumor location**						
Proximal stomach	1		< 0.001			0.404
Distal stomach	0.549	0.434–0.694	< 0.001			0.252
Full stomach	1.118	0.832–1.504	0.459			0.958
PT	1.131	0.913–1.401	0.259			
PTA	0.714	0.552–0.925	0.011			0.782
PTR	1.095	0.871–1.378	0.436			
INR	1.57	1.116–2.209	0.01			0.917
APTT	1.162	0.874–1.543	0.301			
TT	0.733	0.587–0.915	0.006			0.235
FIB	1.729	1.388–2.153	< 0.001			0.214
D-dimer	2.486	1.982–3.118	< 0.001	1.633	1.178–2.264	0.003
FDP	1.838	1.241–2.724	0.002			0.969
Platelet	1.023	0.743–1.41	0.453			
LNR	9.42	6.67–13.303	< 0.001	4.707	2.886–7.677	< 0.001
Tumor size	1.158	1.119–1.198	< 0.001	1.085	1.032–1.141	0.002
**Histology**						
Differentiated	1					
Bndifferentiated	1.055	0.841–1.324	0.641			
**TNM stage**						
I	1		< 0.001	1		< 0.001
II	2.631	1.618–4.277	< 0.001	4.063	1.897–8.705	0.001
III	6.166	4.15–9.161	< 0.001	9.296	4.669–18.51	< 0.001
Chemotherapy^1^	2.163	1.689–2.769	< 0.001	0.42	0.286–0.615	< 0.001

HR, hazard ratio; CI, confidence interval; PT, prothrombin time; PTA, prothrombin activity; PTR, prothrombin ratio; APTT, activated partial prothrombin time; INR, international normalized ratio; TT, thrombin time; FIB, fibrinogen; FDP, fibrin degradation products; TNM, Tumor-node-metastasis; LNR, Lymph node ratio. Coagulation parameters were divided into two groups according to the cutoff values in Supplementary Table 2. The references of parameters were female, age < 65 years, PT < 13.2, PTA < 102.6, PTR < 1, INR < 0.96, APTT < 38.9, TT < 16.5, FIB < 3.8, D-dimer < 1, FDP < 2.1 and Platelet < 300. LNR and tumor size is analyzed as a continuous variable in univariate and multivariate analyses. ^1^This refers to adjuvant chemotherapy, the reference was no chemotherapy; adjuvant chemotherapy appeared to be a risk factor in univariate analysis because cox regression analysis included patients with TNM stage I who did not receive adjuvant chemotherapy.

### Survival analysis of two patient groups after PSM

To eliminate the influence of other covariates on D-dimer prognostic analysis, PSM using a one-to-one nearest neighbor matching method was implemented. After matching, 161 patients in NDG were matched to EDG after adjusting age, gender, TNM stage, tumor location and histology (Fig. 1). There was no significant difference in gender, age, PTA, PTR, INR, TT, APTT, TNM stage, histology and LNR in matched series. Patients in EDG still had shorter OS (median: 19 months vs 25 months, p = 0.005) and shorter DFS (median: 18.5 months vs 24 months, p = 0.007) (Table 3). To our surprise, Spearman correlation analysis indicated a relationship between D-dimer and tumor size (P = 0.002) and N stage (0.011) (see Supplementary Table S4 online). Whereas age (P = 0.053), T stage (P = 0.117) and TNM stage (P = 0.056) showed no correlation with D-dimer. After PSM, the 5-year OS rate in EDG was still shorter than in NDG (32.8% vs 40.6%, p = 0.005) (Fig. 2B), so was DFS (29% vs 36.6%, p = 0.008) (Fig. 3B).

**Table 3:** Demographic and baseline characteristics of the two patient groups after PSM (N = 322).

Characteristics	Total	NDG	EDG	P value
	(N = 322)	(N = 161)	(N = 161)	
**Gender, no. (%)**				0.211
Female	88(27.3)	39(24.2)	49(30.4)	
Male	234(72.7)	122(75.8)	112(69.6)	
**Tumor location, no. (%)**				0.011
proximal stomach	131(40.7)	78(48.4)	53(32.9)	
distal stomach	145(45)	60(37.3)	85(52.8)	
most or total stomach	46(14.3)	23(14.3)	23(14.3)	
Age ≥ 65 years. No. (%)	157(48.8)	78(48.4)	79(49.1)	0.911
PT, second	13(12.4–13.53)	12.9(12.35–13.4)	13.1(12.6–13.7)	0.02
PTA, %	94.1(82.9–105.45)	94.45(86.2–108.5)	94.9(78.9–104.7)	0.142
PTR	1.03(0.99–1.08)	1.03(0.99–1.07)	1.02(0.99–1.08)	0.942
APTT, second	34.6(31.7–37)	1.02(0.98–1.07)	1.03(1–1.09)	0.669
INR	1.03(0.99–1.08)	34.5(32–37.2)	34.7(31.55–36.95)	0.086
TT, second	16.2(15.7–16.8)	16.2(15.75–16.75)	16.2(15.6–16.85)	0.953
FIB, g/l	3.35(2.71–4)	3.18(2.7–3.8)	3.44(2.81–4.2)	0.04
D-dimer, mg/l	1.01(0.3–1.71)	0.3(0.1–0.6)	1.7(1.2–2.75)	< 0.001
FDP, mg/l	1.62(0.9–3.2)	1(0.6–1.3)	3.2(2.3–5.4)	< 0.001
Platelet	199(153–252)	198(151–255.5)	200(155.8–250.5)	0.8
**T stage, no. (%)**				0.737
T1	33(10.2)	15(9.3)	18(11.2)	
T2	20(6.2)	12(7.5)	8(5)	
T3	81(25.2)	42(26.1)	39(24.2)	
T4	188(58.4)	92(57.1)	96(59.6)	
**N stage, no. (%)**				0.01
N0	91(28.3)	40(24.8)	51(31.7)	
N1	50(15.5)	29(18)	21(13)	
N2	77(23.9)	49(30.4)	28(17.4)	
N3	104(32.3)	43(26.7)	61(37.9)	
**TNM stage, no. (%)**				0.057
I	41(12.7)	17(10.6)	24(14.9)	
II	53(16.5)	34(21.1)	19(11.8)	
III	228(70.8)	110(68.3)	118(73.3)	
**Chemotherapy**				0.204
yes	238(73.9)	114(70.8)	124(77)	
no	84(26.1)	47(29.2)	37(23)	
**Histology, no. (%)**				0.051
differentiated	123(38.2)	70(43.5)	53(32.9)	
undifferentiated	199(61.8)	91(56.5)	108(67.1)	
OS, month	24(13–44)	25(15–47.5)	19(10–42.4)	0.005
DFS, month	23(12–44)	24(14–48)	18.5(9–43.25)	0.007
LNR	0.21(0–0.44)	0.21(0–0.36)	0.21(0–0.57)	0.921
Tumor size, cm	4(3–5.5)	3(2.5–4)	4.5(3–6)	< 0.001

## Discussion

In our present study, we investigated the prognostic value of plasma D-dimer for long term survival of GC patients with radical gastrectomy. Our findings indicated that elevated plasma D-dimer was independently associated with poorer OS and DFS. Preoperative D-dimer may provide clinicians with convenient and inexpensive clinical decision-making guidance for patients undergoing radical gastrectomy.

Patients with tumor load are often in a hypercoagulable state. Tumor progression and hypercoagulable state complement each other. Domenico Russo et al. reported that tumor cells as well as host cells which are influenced by local cancer-related inflammatory response express high level of tissue factors (TF) which in turn promoted hypercoagulation^19^. Consistently, Guangjun Nie et al. elucidated that targeting tumor-specific TF remarkably suppress hypercoagulable state^20^. Hypercoagulable state not only increases the risk of thrombotic disease but also promotes tumor progression. Tumor cells promote hypercoagulability through complex mechanisms and then promote self-progress through hypercoagulation. In fact, the risk of a venous thromboembolism is 4- to sevenfold higher in patients with cancer than in those without which is known as Trousseau's syndrome^21^. As part of the coagulation pathway, the platelet/fibrin (ogen) axis has been shown to promote metastasis by preventing natural killer (NK) cells from clearing newly formed micrometastases^22^. Specific thrombin inhibitors, or genetically-mediated decrease in prothrombin expression, significantly limit metastasis^23^. This confirms that tumor progression and hypercoagulability are mutually reinforcing. Recent studies have shown that long-term anticoagulation with warfarin is strongly associated with reduced incidence of various cancers^24^. Therefore, we inferred that the level of coagulation markers may reflect the progress of the tumor. D-dimer is produced when cross-linked fibrin is degraded by plasmin induced fibrinolytic activity. Studies about D-dimer and GC prognosis have been undertaken in many tumor diseases^11,17^. Cihan Ay et al. illuminated that high D-dimer levels were associated with poor OS and increased mortality risk in cancer patients^25^. Long Liu et al. reported that high D-dimer level may predict poor outcome of GC patients^14^. Controversially, Yuexiang Liang et al. illuminated that preoperative D-dimer was not an independent prognostic factor for patients with GC after curative resection^18^. Although the difference in research results may be due to regional differences in distribution and the influence of confounding factors, it is significant to explore the prognostic value of D-dimer in GC.

Our previous studies focused on the effect of D-dimer on short-term survival in GC patients and our current research reviewed 907 GC patients with radical gastrectomy and followed them up for up to 10 years. To detect possible confounding factors, we conducted multivariate analysis and found that elevated plasma D-dimer was an independent risk factor for OS and DFS. To eliminate the influence of interference factors on survival analysis, we performed PSM analysis to adjust age, gender, TNM stage, tumor location and histology. After PSM, the difference of age, gender, TNM stage, and histology were no longer significant but the difference of 5-year OS rate between two groups still remained (EDG vs NDG: 32.8% vs 40.6%, p = 0.005). The 5-year DFS rate of NDG (36.6%) was also significantly higher than EDG (29%) (P = 0.008). This revealed that D-dimer may not only be a marker of coagulation and but also a marker of GC progression. Cancer-associated coagulation disorder is often accompanied by thrombocytosis, hyperfibrinogenemia and D-dimer elevation. It was reported that preoperative hyperfibrinogenemia was an unfavorable prognostic factor for OS in patients with GC^15^. However, our results demonstrated that fibrinogen is not an independent prognostic factor for GC. As one of the hallmarks of hypercoagulable state, it is not surprising that fibrinogen is associated with tumor progression. However, after balancing other coagulation indicators, we found that only elevated D-dimer was an independent risk factor for GC. Some studies have reported that thrombocytosis functioned as an independent prognostic factor for GC patients^26–28^. In general, the interaction between the coagulation system and tumor cells is closely related to thrombosis and tumor progression. Elevated levels of circulating TF have been reported in tumor patients. TF-mediated thrombin generation mediates platelet activation that is crucial for hypercoagulation in malignancy^29^. Platelets contain both pro- and antiangiogenic factors, but platelets and platelet adhesion support angiogenesis^30^. Because platelets secrete factors including matrix metalloproteinases, platelet factor 4 and VEGF, tumors may use the proangiogenic properties of platelets for the formation of new blood vessels, supporting the migration and the activation of endothelial cells^31^. However, we did not find an association between platelets and gastric cancer survival. Tumor promotes hypercoagulable state through procoagulant substances. While a procoagulant milieu supports tumor immune escape and interferes with immune therapy^32^. It seems that anticoagulation therapy may has a positive effect on tumors. In fact, Low-molecular-weight heparin (LMWH) can prolong survival in cancer patients with or without deep vein thrombosis^33^. The risk of venous thromboembolism (VTE) in cancer patients is significantly higher than that in normal people. For cancer-associated VTE, full-dose oral Xa inhibitors have better efficacy than LMWH ^34^. In other words, anticoagulation therapy can not only inhibit tumor progression, but also reduce the risk of tumor-related thrombotic diseases. However, LMWH may have some benefit in limiting cancer progression, but this benefit appears to depend on cancer type, cancer stage, and the specific formulation of LMWH^35^. Moreover, the anticoagulation strategies of tumor patients in different periods and how to synergize with traditional treatment methods need to be further studied. Whether D-dimer can be used as a marker of therapeutic effect also need to be explored.

Retrospective nature was one limitation of our study. Although we enrolled clinical data from three tertiary referral centers, but they are located in northwest China, which may cause bias. On the other hand, the effect of postoperative radiotherapy on survival analysis was not taken into consideration in this study.

## Conclusions

Elevated preoperative D-dimer levels is closely associated with older age, advanced clinical pathological stage, larger tumor size and more lymph node metastasis. It’s also identified as an independent prognostic factor for GC patients after radical gastrectomy.

## Materials and methods

### Patients

We retrospectively enrolled 903 GC patients who underwent radical gastrectomy between January 2009 and February 2017. Disease clinical staging (I-IV) depended on the eighth edition systems recommended by American Joint Committee on Cancer (AJCC). Patients follow up data were obtained by regular follow-up. The date of final follow-up was in June 2021. The interval between the dates of radical surgery and either the time of the last follow-up or the time of death was defined as OS, between the dates of radical survival and either the time of the last follow-up or the time of relapse was defined as DFS. For OS, the endpoint event was any cause of death. For DFS, the endpoint event was disease recurrence, death or secondary tumor. Censoring meant that no endpoint event was observed at the last follow-up. Inclusion criteria: (1) all patients were newly diagnosed with GC; (2) all patients were pathologically diagnosed; (3) all patients had pre-treatment coagulation test; (4) stage I-III disease; (5) age ≥ 18 years; (6) Radical treatment and R0 resection. Exclusion criteria: (1) accompanying or secondary to other tumors; (2) had history of venous thrombosis or received any anti-coagulation treatment; (3) acute infection or diffuse intravascular coagulation; (4) pregnancy or lactation; (5) history of neoadjuvant chemotherapy; (6) Lost to follow-up. Pre-treatment coagulation indicators was those closest to the time of treatment. This study was approved by the Ethics Committee of First Affiliated Hospital of Xi'an Jiaotong University. Informed consent was obtained from all subjects and/or their legal guardian(s). All methods were performed in accordance with the relevant guidelines and regulations.

### Coagulation index assay

Venous blood was collected in sodium citrate tubes. The levels of FIB, FDP and D-dimer were analyzed by latex-enhanced immunoturbidimetric assay. All the samples were collected before any treatments. The normal level of FIB, D-Dimer and FDP in human plasma is less than 4.0 g/l, 1.0 mg/l and 5.0 mg/l, respectively.

### Evaluation of baseline characteristics

We collected gender, age at surgery, pre-treatment laboratory test [including prothrombin time (PT), prothrombin activity (PTA), prothrombin ratio (PTR), activated partial prothrombin time (APTT), international normalized ratio (INR), thrombin time (TT), fibrinogen (FIB), D-dimer, fibrin degradation products (FDP)], T stage, N stage, Tumor-node-metastasis (TNM) stage, histology, OS, DFS, lymph nodes retrieval and tumor size. Lymph node ratio (LNR) was calculated by dividing the number of positive lymph nodes by the number of resected lymph nodes. We focused on the effect of hypercoagulability on tumor, so we set the upper limit of normal range of coagulation parameters as the cut-point. We classified tumors into two groups based on histology: undifferentiated type (including undifferentiated or poorly differentiated adenocarcinoma, mucinous carcinoma and signet ring cell carcinoma), differentiated type (including well or moderately differentiated adenocarcinoma).

### Statistical analysis

The optimal cutoff vale of parameters was obtained by receiver operating characteristic curve (ROC). The cutoff value of age was set to 65 years. Cases were divided into normal D-dimer group (NDG) and elevated D-dimer group (EDG) based on cutoff value. Categorical variates were presented as frequencies and percentages and compared using the chi-square test or Fisher exact test. Continuous non-normal variates were presented as the median and interquartile range (IQR) and compared with log-rank tests, while continuous normally distributed variates were presented as mean and standard deviation and compared by Student's *t-tests*. Differences of OS and DFS were assessed by the log-rank test and visualized using the Kaplan–Meier method. We divided age, PT, PTA, PTR, INR, APTT, TT, FIB, D-dimer and FDP into two groups based on cutoff values. Then independent factors of OS and DFS was determined by multivariate Cox regression analyses and assessed by the *Wald* test. Variates with *P* < 0.05 in univariate analysis were included in multivariate analysis. To eliminate the influence of potential confounders on survival analysis, PSM analysis was performed by a one-to-one nearest neighbor matching method. Match tolerance was set to 0.02. The matching factor involved in the propensity model was age, gender, TNM stage, tumor location and histology.

Statistical analysis and plotting were performed with SPSS Statistics (version 22.0, IL, USA), GraphPad Prism v.8.0.1 (La Jolla, CA, USA). X-tile 3.6.1 software^36^ (Yale University, New Haven, CT, USA). 2-sided *p* < 0.05 were considered statistical significantly.

## Supplementary Information

Supplementary Information.

## Data Availability

The data generated in this study are available upon request from the corresponding author.
