# Supplementary material for: D-dimer, a predictor of bad outcome in gastric cancer patients undergoing radical resection
Source: Sci Rep. 2022 Sep 30;12:16432. doi: 10.1038/s41598-022-16582-9 (PMC9525585; doi:10.1038/s41598-022-16582-9)
Supplement: Supplementary file 1 — Supplementary Information. [file 41598_2022_16582_MOESM1_ESM.doc]

**D-dimer, A Predictor of Bad Outcome in Gastric Cancer Patients undergoing radical resection**

Xin Zhang^1M.D.^, Xuan Wang^1 M.D.^, Wenxing Li^1M.D.^, Tuanhe Sun^1M.D.^, Chengxue Dang^#1M.D., Ph.D^. and Dongmei Diao^#1 M.D., Ph.D^.

^1^Department of Oncology Surgery, First Affiliated Hospital of Xi’an Jiaotong University, Xi’an, China;

^#^ Corresponding Author: Dr. Dongmei Diao and Prof. Chengxue Dang, Ph.D., M.D., Department of Surgical Oncology, First Affiliated Hospital Medical college Xi'an Jiaotong University, 277 West Yanta Road, Xi’an, Shaanxi 710061, P.R. China. Tel: 86-29-85324612, Fax: 86-29-85324612, E-mail: [diaomei310@mail.xjtu.edu.cn](mailto:diaomei310@mail.xjtu.edu.cn); [dangchengxue@mail.xjtu.edu.cn](mailto:dangchengxue@mail.xjtu.edu.cn).

**Supplemental Figure 1.**


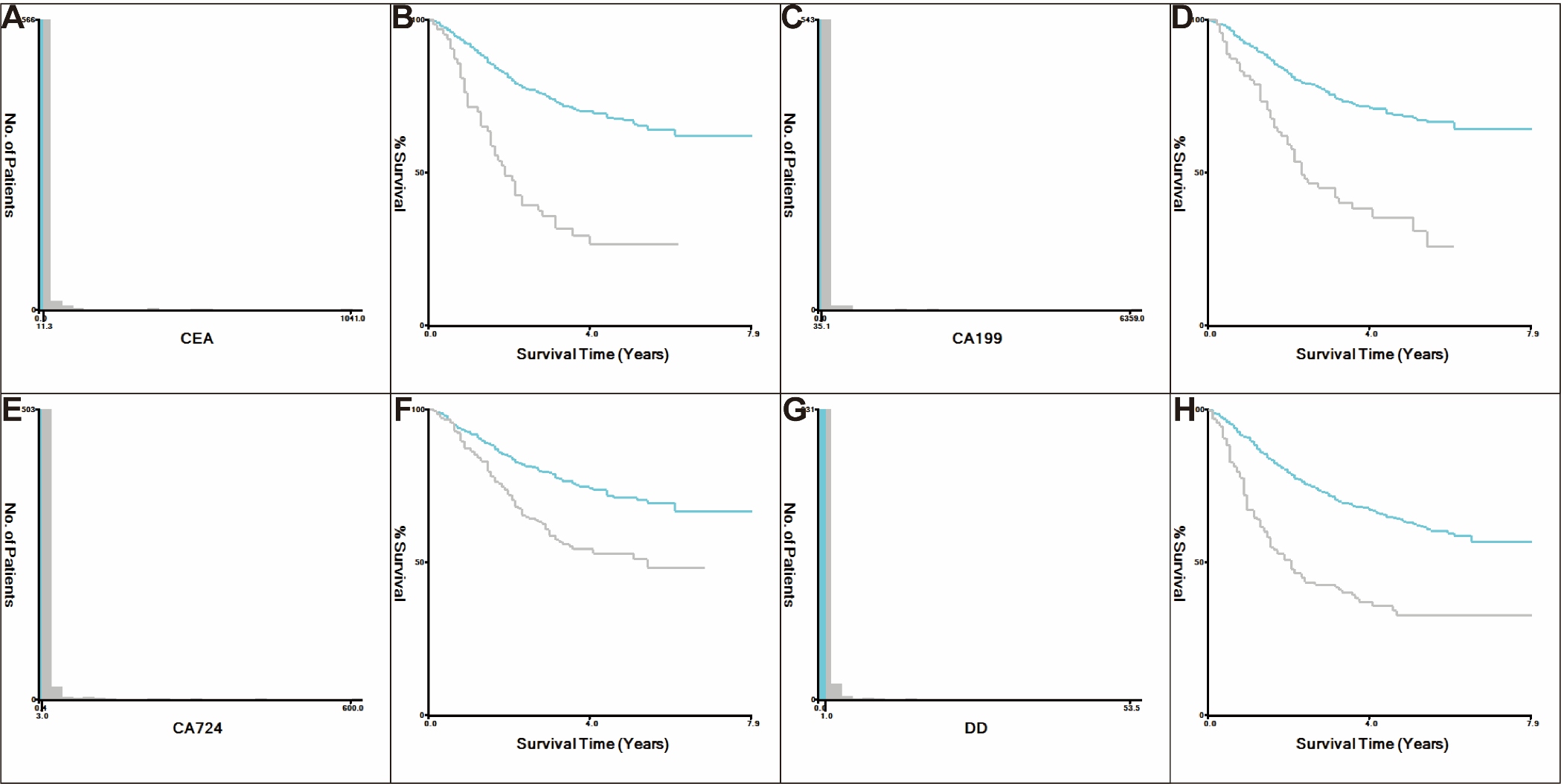


**Supplemental Figure 1. Cutoffs and Survival Curves.** (A-B) for CEA, (C-D) for CA19-9, (E-F) for CA72-4 and (G-H) for D-dimer.

**Supplemental Figure 2.**


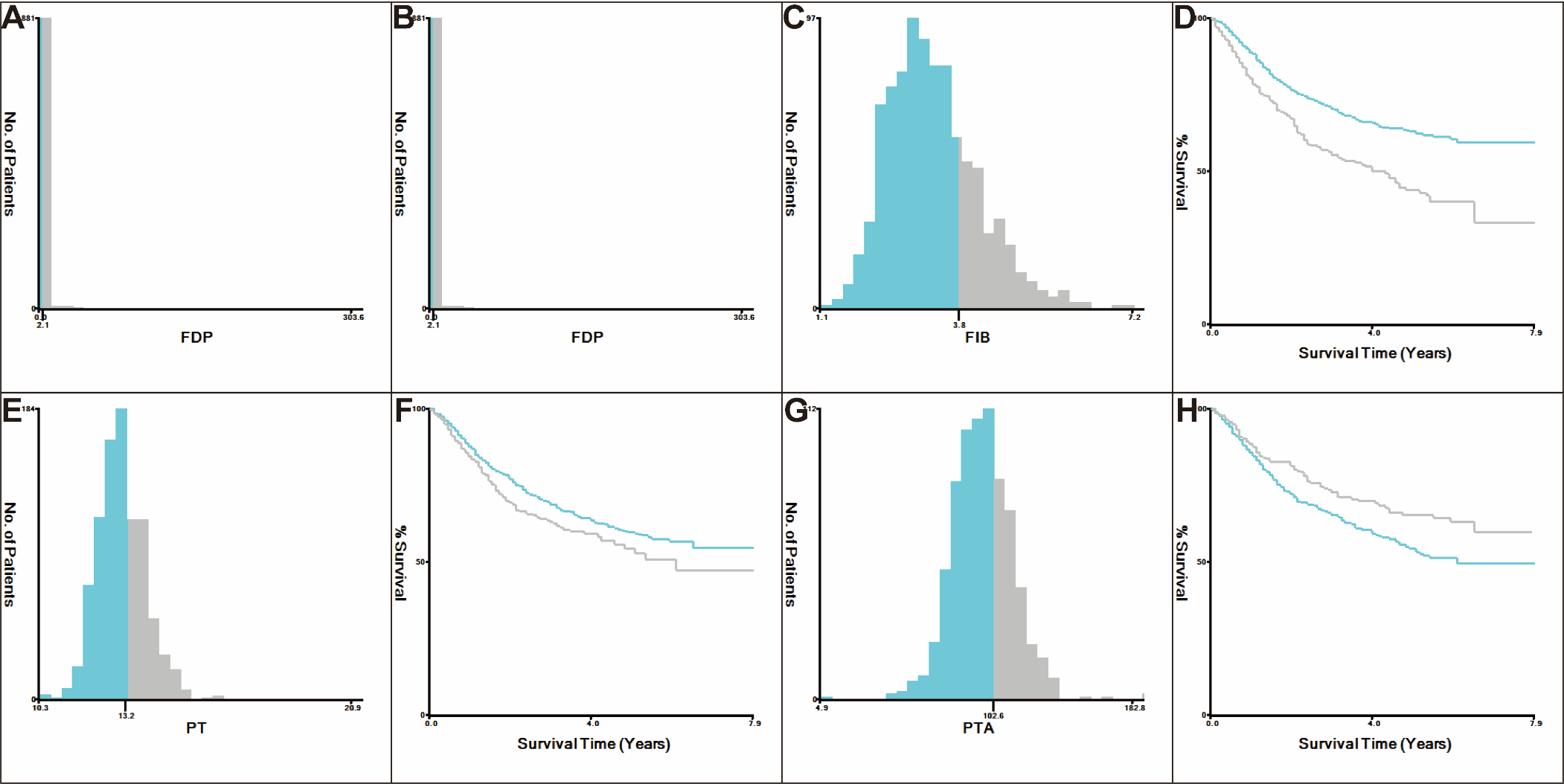


**Supplemental Figure 2. Cutoffs and Survival Curves.** (A-B) for FDP, (C-D) for FIB, (E-F) for PT and (G-H) for PTA.

**Supplemental Figure 3.**


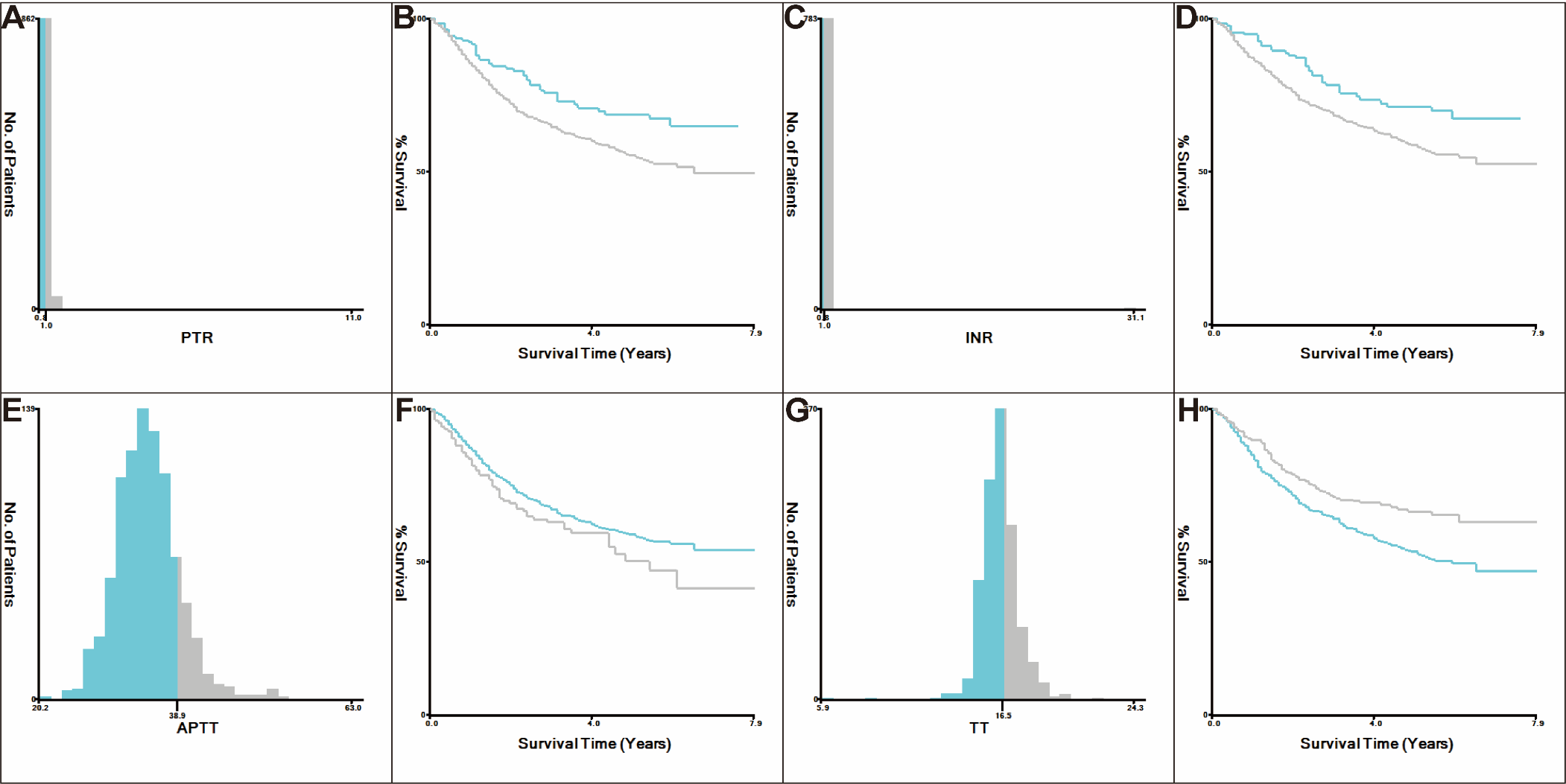


**Supplemental Figure 3. Cutoffs and Survival Curves.** (A-B) for PTR, (C-D) for INR, (E-F) for APTT and (G-H) for TT.

Supplemental Table 1. Correlation between plasma D-dimer level and clinicopathological characteristics before PSM

|  | Spearman correlation | P value |
| --- | --- | --- |
| Age | 0.184 | <0.001 |
| Tumor size | 0.116 | 0.002 |
| T stage | 0.095 | 0.001 |
| N stage | 0.178 | <0.001 |
| TNM stage | 0.270 | <0.001 |

**Supplementary Table 2. The cut-off points** for continuous variables.

| Variable | Cut-off | Miller-Seigmund P | Chi-sp Hi/LO |
| --- | --- | --- | --- |
| PT | 13.2 | 0.5174 | 3.6509 |
| PTA | 102.6 | 0.1269 | 7.2251 |
| PTR | 1 | 0.1023 | 7.7012 |
| INR | 0.96 | 0.1443 | 6.9061 |
| APTT | 38.9 | 1 | 2.3109 |
| TT | 16.5 | 0.0207 | 11.3215 |
| FIB | 3.8 | <0.001 | 24.6556 |
| D-dimer | 1 | <0.001 | 68.1349 |
| FDP | 2.1 | <0.001 | 67.5264 |

**Miller-Seigmund P:** Provides a corrected p-value based on the model proposed by R Miller and D. Siegmund. Chi-sp Hi/LO: Provides the 2-population log-rank chi-square value.

**Supplemental Table 3. Univariate and multivariate analyses for disease-free survival of GC patients before PSM (N=903).**

| Parameters | Univariate analysis | | | Multivariate analysis | | |
| --- | --- | --- | --- | --- | --- | --- |
|  | HR | 95%CI | P value | HR | 95%CI | P value |
| Gender, Female vs male | 1.205  1.205 | 0.947-1.533 | 0.13 |  |  |  |
| Age, <65 vs ≥65 | 1.634 | 1.331-2.006 | <0.001 | 1.914 | 1.448-2.531 | <0.001 |
| Tumor location, no. (%) |  |  |  |  |  |  |
| proximal stomach | 1 |  | <0.001 |  |  | 0.305 |
| distal stomach | 0.544 | 0.435-0.679 | <0.001 |  |  | 0.217 |
| full stomach | 1.05 | 0.788-1.398 | 0.74 |  |  | 0.849 |
| PT | 1.164 | 0.949-1.427 | 0.144 |  |  |  |
| PTA | 0.736 | 0.576-0.941 | 0.015 |  |  | 0.918 |
| PTR | 1.157 | 0.928-1.443 | 0.195 |  |  |  |
| INR | 1.556 | 1.124-2.155 | 0.008 |  |  | 0.885 |
| APTT | 1.097 | 0.833-1.445 | 0.51 |  |  |  |
| TT | 0.745 | 0.603-0.92 | 0.006 |  |  | 0.181 |
| FIB | 1.68 | 1.362-2.074 | <0.001 |  |  | 0.372 |
| D-dimer | 2.506 | 2.018-3.112 | <0.001 | 1.58 | 1.151-2.169 | 0.005 |
| FDP | 1.997 | 1.382-2.884 | <0.001 |  |  | 0.746 |
| Platelet | 0.989 | 0.726-1.347 | 0.943 |  |  |  |
| LNR | 9.237 | 6.631-12.865 | <0.001 | 4.865 | 3.037-7.793 | <0.001 |
| Tumor size, cm | 1.157 | 1.119-1.196 | <0.001 | 1.084 | 1.034-1.141 | 0.001 |
| Histology |  |  |  |  |  |  |
| differentiated | 1 |  |  |  |  |  |
| undifferentiated | 1.035 | 0.834-1.283 | 0.756 |  |  |  |
| TNM stage |  |  |  |  |  |  |
| I | 1 |  | <0.001 | 1 |  | <0.001 |
| II | 2.482 | 1.59-3.8874 | <0.001 | 3.615 | 1.792-7.293 | <0.001 |
| III | 5.568 | 3.883-7.982 | <0.001 | 7.803 | 4.136-14.722 | <0.001 |
| Chemotherapy^1^ | 2.175 | 1.719-2.751 | <0.001 | 0.434 | 0.3-0.63 | <0.001 |

HR, hazard ratio; CI, confidence interval; PT, prothrombin time; PTA, prothrombin activity; PTR, prothrombin ratio; APTT, activated partial prothrombin time; INR, international normalized ratio; TT, thrombin time; FIB, fibrinogen; FDP, fibrin degradation products; TNM, Tumor-node-metastasis; LNR, Lymph node positive rate. Coagulation parameters were divided into two groups according to the cutoff values in Supplementary Table 2. The references of parameters were female, age<65 years, PT<13.2, PTA<102.6, PTR<1, INR<0.96, APTT<38.9, TT<16.5, FIB<3.8, D-dimer<1, FDP<2.1 and Platelet<300. LNR and tumor size is analyzed as a continuous variable in univariate and multivariate analyses. ^1^Refers to adjuvant chemotherapy.

Supplemental Table 4. Correlation between plasma D-dimer level and clinicopathological characteristics after PSM

|  | Spearman correlation | P value |
| --- | --- | --- |
| Age | 0.108 | 0.053 |
| Tumor size | 0.268 | 0.002 |
| T stage | 0.088 | 0.117 |
| N stage | 0.141 | 0.011 |
| TNM stage | 0.107 | 0.056 |

**Supplemental Table 5. Univariate and multivariate analyses for overall survival of GC patients before PSM (N=322).**

| Parameters | Univariate analysis | | | Multivariate analysis | | |
| --- | --- | --- | --- | --- | --- | --- |
|  | HR | 95%CI | P value | HR | 95%CI | P value |
| Gender | 1.586 | 1.116-2.253 | 0.01 | 1.198 | 1.112-3.532 | 0.02 |
| Age | 1.206 | 0.897-1.623 | 0.215 |  |  |  |
| Tumor location |  |  |  |  |  |  |
| proximal stomach | 1 |  | 0.05 |  |  |  |
| distal stomach | 0.784 | 0.563-1.093 | 0.152 |  |  |  |
| full stomach | 1.293 | 0.852-1.963 | 0.228 |  |  |  |
| PT | 1.425 | 1.057-1.921 | 0.02 | 2.011 | 1.238-3.265 | 0.005 |
| PTA | 0.826 | 0.61-1.12 | 0.219 |  |  |  |
| PTR | 0.952 | 0.683-1.328 | 0.774 |  |  |  |
| INR | 1.387 | 0.753-2.557 | 0.294 |  |  |  |
| APTT | 0.986 | 0.64-1.517 | 0.947 |  |  |  |
| TT | 0.834 | 0.61-1.141 | 0.256 |  |  |  |
| FIB | 1.547 | 1.142-2.095 | 0.005 | 1.66 | 1.036-2.661 | 0.035 |
| D-dimer | 1.513 | 1.122-2.041 | 0.007 |  |  |  |
| FDP | 1.518 | 1.128-2.044 | 0.006 |  |  |  |
| Platelet | 0.994 | 0.628-1.573 | 0.981 |  |  |  |
| LNR | 3.543 | 2.006-6.255 | <0.001 |  |  |  |
| Tumor size | 1.127 | 1.034-1.228 | 0.007 |  |  |  |
| Histology | 0.905 | 0.664-1.235 | 0.529 |  |  |  |
| TNM stage |  |  |  |  |  |  |
| I | 1 |  | <0.001 |  |  |  |
| II | 2.019 | 0.945-4.315 | 0.07 |  |  |  |
| III | 3.897 | 2.049-7.411 | <0.001 |  |  |  |
| Chemotherapy | 0.856 | 0.615-1.191 | 0.356 |  |  |  |
